# Uterine perforation during uterine repositioning on nonpuerperal uterine inversion by delivery of a large fibroid: A case report

**DOI:** 10.1002/ccr3.7810

**Published:** 2023-08-23

**Authors:** Mari Uomoto, Aya Tokinaga‐Uchiyama, Natsuko Kobayashi, Mika Okuda

**Affiliations:** ^1^ Department of Obstetrics and Gynecology National Hospital Organization Yokohama Medical Center Yokohama Japan

**Keywords:** fibroma, laparoscopy, nonpuerperal uterine inversion, perforation

## Abstract

**Key Clinical Message:**

Transvaginal uterine restoration of nonpuerperal uterine inversion is difficult; there are risks of heavy bleeding and uterine perforation. In such cases, total hysterectomy and transfusion are inevitable.

**Abstract:**

A 47‐year‐old woman with profuse genital bleeding, diagnosed with nonpuerperal uterine inversion caused by a uterine fibroma, underwent emergency surgery. Uterine perforation occurred during transvaginal uterine restoration, revealed by laparoscopy. Bleeding persisted and blood transfusion volume increased; therefore, a total hysterectomy was performed for hemostasis.

## Graphical abstract

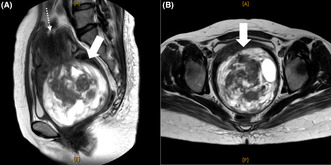

## 1 INTRODUCTION

Uterine inversion can be divided into two major types: puerperal and nonpuerperal, with the latter accounting for 17% of all uterine inversion cases.^1^ The average age of patients with uterine inversion is 46.3 years, and affected patients often complain of abdominal pain and irregular bleeding.^2^ The majority of cases are caused by uterine myoma, with rare cases due to malignant uterine cancer or sarcoma.^1^, ^2^ Meanwhile, the characteristic magnetic resonance imaging (MRI) findings are a V‐shaped depressed uterine fundus and a tumor showing a bull's‐eye sign in the vaginal cavity.^3^ Uterine repositioning on nonpuerperal uterine inversion has risks of uterine perforation and heavy bleeding resulting in severe blood loss.^4^ However, these risks are poorly studied. Here, we experienced an acute case of nonpuerperal uterine inversion in a 47‐year‐old woman and attempted uterine restoration under laparoscopic observation.

## 2 CASE PRESENTATION

**FIGURE 1:**
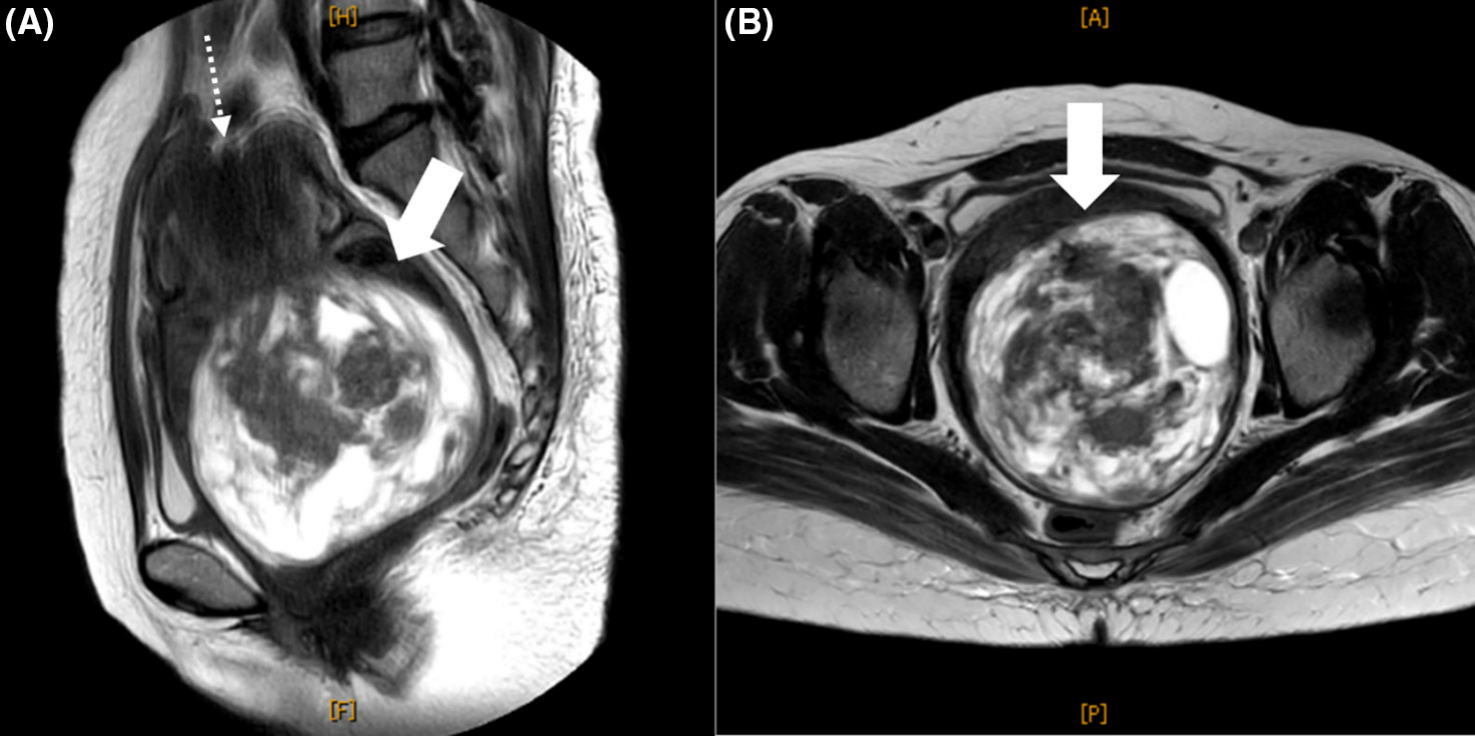
Magnetic resonance imaging showing uterine inversion caused by fibroid delivery. (A) A 10‐cm‐sized nodule in the vagina and “V shape” uterine fundus. (B) Layers of the inverted uterus with “bull's‐eye” configuration.

**FIGURE 2:**
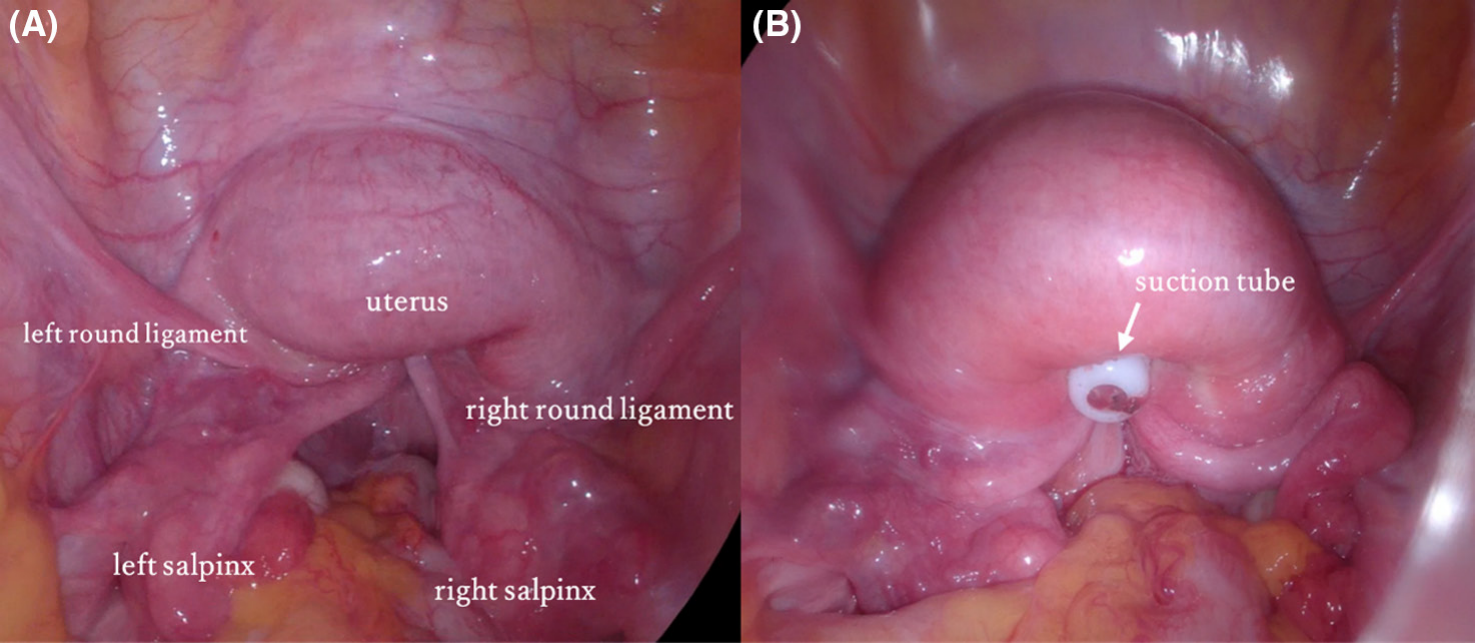
Laparoscopic imaging. *Arrows* indicate nodules in each panel. *An arrow with dots* indicates the inverted uterine fundus. (A) Uterus inversion. Bilateral round ligaments and salpinx are included in the uterine fundus. (B) Uterus perforation during trial of uterus reposition with suction tube.

A 47‐year‐old sexually inexperienced woman who had been aware of excessive menstruation for several years visited her local gynecology department 1 month prior, with the chief complaint being excessive menstruation. At that time, she weighed 60 kg and was 157 cm tall. She was conscious, and her blood pressure was 110/78 mm Hg; heart rate, 99 bpm; and respiratory rate, 15/min, with oxygen saturation of 99% on room air. General physical examination showed no anomaly except for palpebral conjunctival pallor. Transabdominal ultrasonography showed a mass in the uterus. The doctor recommended gynecological examination; however, the patient rejected it because she was sexually inexperienced. She was administered an iron injection, because her blood test revealed anemia (hemoglobin [Hb] level, 3.3 mg/dL). The doctor made a reservation in our hospital for a more detailed examination the next day; however, the patient did not visit the hospital. One month later, she experienced sudden, profuse, irregular genital bleeding and was brought to our hospital for emergency care. She had no relevant medical history except excessive menstruation. When examined in the emergency department, she was conscious, and her blood pressure was 106/66 mm Hg; pulse rate, 110 bpm; and respiratory rate, 17/min, with oxygen saturation of 99% on room air. Head examination showed palpebral conjunctival pallor, and vaginal examination revealed heavy continuous vaginal bleeding. No physical findings of bleeding tendency, such as subcutaneous bleeding, were noted. Vaginoscopy revealed a 10‐cm‐sized myoma in the vaginal cavity. Simultaneously, we conducted cervical cytology pap smear and endometrial cytology tests. A week later, both tests yielded negative results for malignancy. Transabdominal ultrasonography revealed a 10‐cm‐sized mass in the lower abdomen. Pelvic MRI showed a V‐shaped depression of the uterine fundus and a tumor in the extended vaginal canal with a bull's‐eye sign, with uterine inversion caused by fibroma delivery (Figure 1A,B). The patient had persistent genital bleeding, and blood tests showed anemia, with a Hb level of 6.6 mg/dL. The patient requested uterine preservation and minimally invasive surgery. We decided to perform vaginal myomectomy and uterine repositioning under laparoscopic observation, and she agreed to undergo laparotomy or total hysterectomy, depending on the operative findings. During the operation, laparoscopic observation of the abdominal cavity revealed that the uterine fundus was depressed—including the bilateral round ligaments and fallopian tubes (Figure 2A). Consequently, uterine inversion associated with fibroma delivery was diagnosed. The myoma was removed by transvaginal resection, while observing the intra‐abdominal cavity under laparoscopy. We sutured the fibroma‐resected site with two layers of sutures, which effectively stopped the bleeding. The uterine fundus was then pushed up transvaginally under laparoscopic observation; however, the uterus was not repositioned. Additionally, a uterine perforation was observed during the operation (Figure 2B). Owing to persistent bleeding, we judged that transvaginal repositioning would be difficult. Since uterine manipulator insertion and fixation were not possible due to the uterine perforation and dilation of the cervical os with the delivery of the fibroid, we switched to a laparotomy. The uterus was repositioned using Huntington's procedure so that the bilateral round ligaments were pulled cephalad‐ward, and the perforation site was repaired with two layers of sutures. While observing the peritoneal cavity, we noted that bleeding at the site of perforation had stopped. However, vaginal bleeding recurred, and further blood transfusion was required. Therefore, we performed a total hysterectomy for hemostasis. The intraoperative blood loss was 1760 mL, within the measurable range, and the transfusion volume was 12 units of concentrated red blood cells and 14 units of fresh frozen plasma. The patient had a good postoperative course and was discharged from the hospital on postoperative day 6. The resected tumor was pathologically diagnosed as a fibroma. At follow‐up 6 months after the operation, the patient showed no complications.

## 3 DISCUSSION

This case presents two major findings. First, uterine perforation can occur during transvaginal uterine restoration for nonpuerperal uterine inversion, and observation under laparoscopy can be used for a safer procedure. Second, repair of nonpuerperal uterine inversion is not straightforward—if bleeding is not controlled, a total hysterectomy will be necessary, and the patient must be prepared for surgery with adequate blood transfusions.

Uterine repositioning is complicated in nonpuerperal uterine inversion. There are several known methods of uterine restoration—including manual repositioning, transabdominal methods such as Huntington's and Haultain's procedures, and transvaginal methods such as Spinelli's and Kustener's procedures^5^; however, there are few reports of successful restoration using these methods. Herath et al. reported that only 20/133 (15.0%) patients were successfully treated using these methods.^2^ Silva et al. showed that, of 170 patients with nonpuerperal uterine inversion, 147 (86.8%) underwent total hysterectomy due to failure of uterine restoration or hemostasis.^4^ These data further support the fact that uterine restoration in nonpuerperal uterine inversion is challenging. We found that uterine perforation further poses a risk during transvaginal uterine restoration and is more difficult to recognize in uterine inversion cases than in normal cases. In a previously reported case, an ovary was found in the uterus during transvaginal uterine restoration of a nonpuerperal uterine inversion, and the patient underwent transabdominal surgery due to suspicion of uterine perforation.^6^ Fortunately, in our case, complications, such as intestinal perforation, did not occur. However, there would have been a risk of intestinal perforation if the transvaginal operation had proceeded without the uterine perforation being noticed. In fact, a case of intestinal perforation during a miscarriage surgery—requiring intestinal repair—was reported in a similar vaginal operation.^7^ If the operation is completed without identification of uterine perforation, there is a risk of postoperative pelvic infection or increased bleeding from the perforated site, and thus, reoperation may be required.

In these cases, laparoscopic observation during the repositioning of nonpuerperal uterine inversion allows for a potential perforation to be recognized and the operation to proceed safely by switching to laparotomy at the appropriate time.

Although rare, some cases of nonpuerperal uterine inversion may have an acute course, as in the present case. Acute cases of nonpuerperal uterine inversion account for 8.6% of all such cases.^8^ All reported cases of acute nonpuerperal uterine inversion have been associated with heavy bleeding and hypovolemic shock.^9^, ^10^ In our case, the patient was brought to the emergency room because of sudden, heavy bleeding and was in hypovolemic shock, with a shock index of 1.1 when admitted. Thus, emergency surgery was performed while administering blood transfusion. Moreover, as in the present case, even after achieving hemostasis at the site where the tumor was resected, bleeding from the endometrium may resume after repositioning of the uterus as a result of suture loosening during repositioning. In the repositioning of the uterus for nonpuerperal uterine inversion, a large amount of blood for transfusion may be required. Therefore, before operation, patients should be informed about the possibility of total hysterectomy for hemostasis.

## 4 CONCLUSION

Sufficient blood transfusions should be prepared in case of heavy bleeding before and during surgery; otherwise, the patient should be considered for transfer to a higher‐level medical institution. Additionally, transvaginal uterine restoration is not easy, and there is a risk of heavy bleeding and uterine perforation. If perforation occurs, the manipulator cannot be inserted and fixed, and laparotomy or total hysterectomy may be necessary. In the present case, the patient requested vaginal surgery and uterine preservation; however, after explaining that laparotomy and total hysterectomy may be unavoidable and obtaining consent before surgery, we decided to change the surgical technique at an appropriate time during surgery.

Thus, in acute cases of nonpuerperal uterine inversion, preoperative and intraoperative blood loss may be high, and bleeding may not be controlled. The operation can be made safer by ensuring sufficient transfusions and being prepared for conversion to laparotomy or total hysterectomy preoperatively.

## AUTHOR CONTRIBUTIONS

**Mari Uomoto:** Conceptualization; investigation; project administration; writing – original draft; writing – review and editing. **Aya Tokinaga#x2010;Uchiyama:** Supervision; writing – review and editing. **Natsuko Kobayashi:** Resources; visualization; writing – review and editing. **Mika Okuda:** Supervision; writing – review and editing.

## FUNDING INFORMATION

No financial support was received for this case report.

## CONFLICT OF INTEREST STATEMENT

The authors declare that they have no conflicts of interest.

## CONSENT

Written informed consent was obtained from the patient to publish this report in accordance with the journal's patient consent policy.

## Data Availability

Data sharing not applicable to this article as no datasets were generated.
